# Endothelial AHR activity prevents lung barrier disruption in viral infection

**DOI:** 10.1038/s41586-023-06287-y

**Published:** 2023-08-16

**Authors:** Jack Major, Stefania Crotta, Katja Finsterbusch, Probir Chakravarty, Kathleen Shah, Bruno Frederico, Rocco D’Antuono, Mary Green, Lucy Meader, Alejandro Suarez-Bonnet, Simon Priestnall, Brigitta Stockinger, Andreas Wack

**Affiliations:** 1 Immunoregulation Laboratory, Francis Crick Institute, London, UK; 2 Bioinformatics, Francis Crick Institute, London, UK; 3 AhRimmunity Laboratory, Francis Crick Institute, London, UK; 4 Immunobiology Laboratory, Francis Crick Institute, London, UK; 5 Light Microscopy, Francis Crick Institute, London, UK; 6 Experimental Histopathology, Francis Crick Institute, London, UK; 7 Department of Pathobiology & Population Sciences, Royal Veterinary College, Hertfordshire, UK

## Abstract

Disruption of the lung endothelial-epithelial cell barrier following respiratory virus infection causes cell and fluid accumulation in the air spaces and compromises vital gas exchange function^1^. Endothelial dysfunction is known to exacerbate tissue damage^2,3^, yet it is unclear whether the lung endothelium promotes host resistance against viral pathogens. Here we show that the environmental sensor aryl hydrocarbon receptor (AHR) is highly active in lung endothelial cells and protects against influenza-induced lung vascular leakage. Loss of AHR in endothelia exacerbates lung damage and promotes infiltration of red blood cells and leukocytes into alveolar air spaces, compromises barrier protection, and increases host susceptibility to secondary bacterial infections. AHR engages tissue-protective transcriptional networks in endothelia, including the vasoactive apelin/APJ peptide system^4^, to prevent a dysplastic and apoptotic response in airway epithelial cells. Finally, we show that protective AHR signalling in lung endothelial cells is dampened by the infection itself. Maintenance of protective AHR function requires a diet enriched in naturally occurring AHR ligands, which activate disease tolerance pathways in lung endothelia to prevent tissue damage. Our findings demonstrate the importance of endothelial function in lung barrier immunity. We identify a gut-lung axis which affects lung damage upon encounter with viral pathogens, linking dietary composition and intake to host fitness and inter-individual variations in disease.

Historically, the host response to infection at barrier surfaces has largely been studied through the lens of immune cell behaviour and function. Only recently have research efforts begun to shed light on the role of non-immune cells in orchestrating tissue immunity upon pathogen encounter^5–8^. This includes the pulmonary endothelium, a monolayer of endothelial cells whose primary function is mediating oxygen intake and delivery. In response to lung challenge, the pulmonary endothelium proliferates^9,10^, regulates proinflammatory cytokine production^3^, and promotes epithelial recovery via secretion of angiocrine factors^11–13^, yet, it is unclear how transcriptional programmes are regulated in lung endothelia to respond to infection or promote host protection.

AHR is a ligand-activated transcription factor widely expressed at barrier sites and in the immune system, poised to respond to physiological and environmental stimuli^14^. Natural AHR ligands largely derive from dietary indoles and tryptophan metabolism^15^. Upon activation, AHR induces a battery of target genes, including cytochrome p450 (CYP1) enzymes: CYP1A1, CYP1B1, and CYP1A2, which promote the metabolic clearance of AHR ligands through oxidation. CYP1-mediated ligand metabolism therefore represents an important negative feedback mechanism that curtails AHR signalling^16,17^. AHR effects are best characterised in the gastrointestinal tract, where abundant microbial- and dietary-derived ligands activate tissue-resident immune cells and epithelia to promote barrier protection^18–24^. In the lung, AHR negatively regulates the production of type I interferons (IFN)^25^, promotes multiciliogenesis in airway epithelial cells^26^, and contributes to bacterial sensing^27^; however, the role of AHR in lung barrier defence is unclear.

## AHR signalling in the lung vasculature

**Extended Data Fig. 1:**
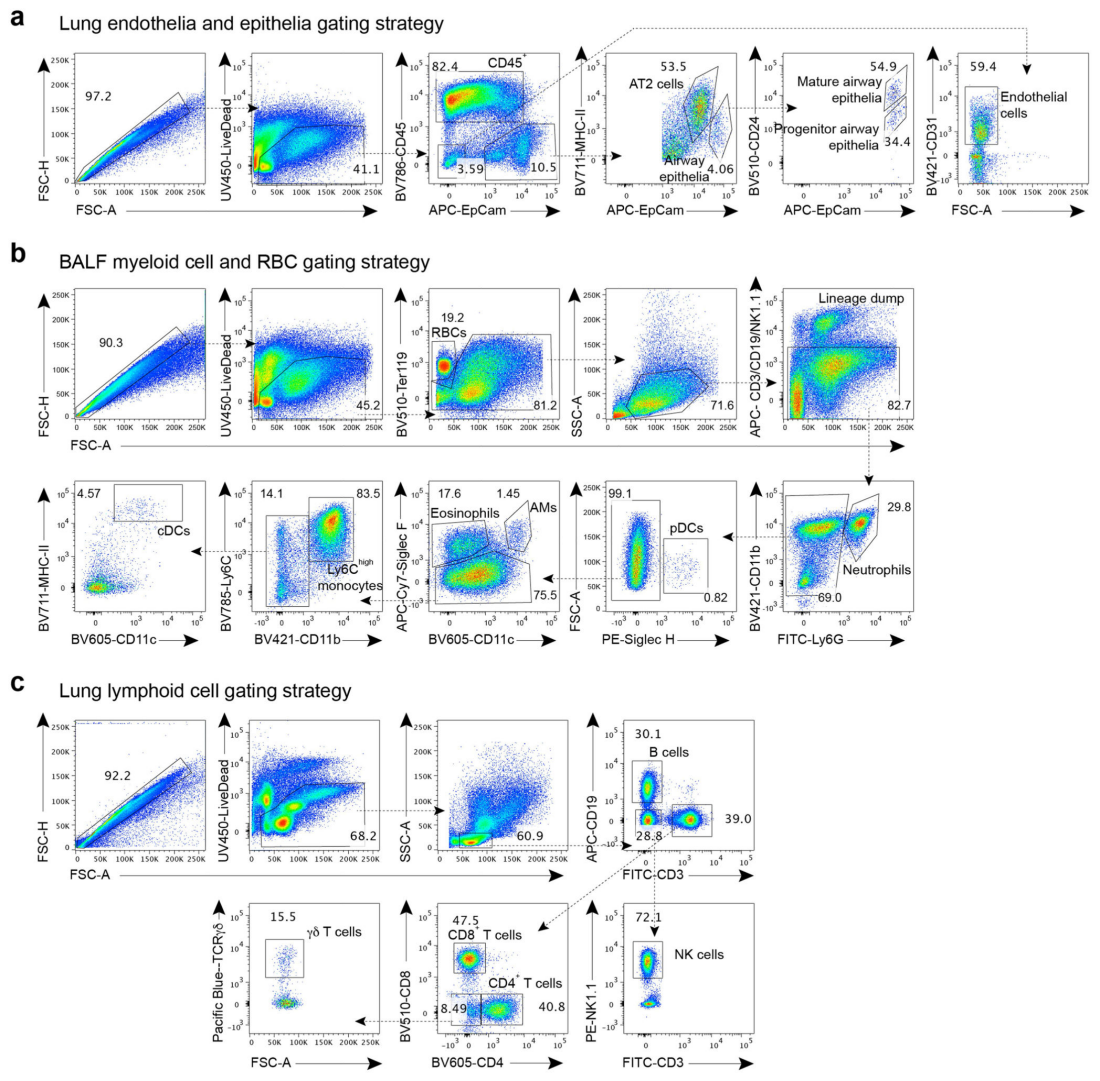
Gating strategies for lung cell populations. **a-c,** Gating strategy for lung endothelial cell and epithelial cell populations (**a**), RBCs and immune cells in the BALF day 6 post influenza virus infection (**b**), and lung lymphoid immune cell populations (**c**) analysed by flow cytometry.

**Extended Data Fig. 2:**
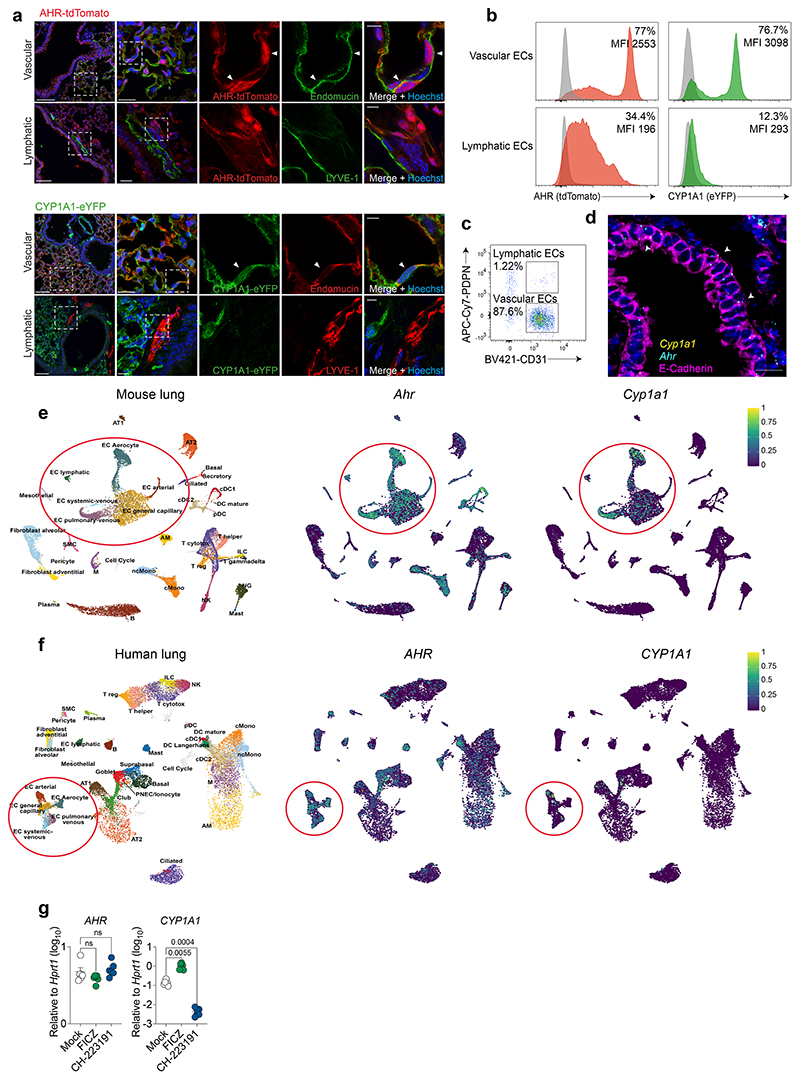
The AHR landscape in mouse and human lung endothelia. **a,** Immunofluorescence staining of steady-state AHR-tdTomato and Cyp1a1-eYFP lung sections stained with the vascular endothelial marker endomucin or lymphatic marker LYVE-1, and Hoechst (blue). Scale bars, 100 μm (left panels), 20 μm (middle panels), 5 μm (right panels). Data are representative of three independent experiments with similar results. **b,** Representative histogram plots of AHR-tdTomato and Cyp1a1-eYFP expression in steady-state lung endothelial cells (CD31^+^PDPN^–^) and lymphatic endothelia (CD31^+^PDPN^+^) relative to B6 WT controls (grey). Mean fluorescence intensity (MFI). **c** Frequency of lymphatic and vascular endothelial cells measured by flow cytometry. **d,** RNA-FISH analysis of WT steady-state lung. RNA probes for *Ahr* (cyan) and *Cyp1a1* (yellow) and stained with E-Cadherin for epithelia. White arrowheads indicate *Ahr* expression in airway epithelial cells. Scale bar, 20 μm. Data are representative of four independent experiments with similar results. **e, f,** Expression of indicated genes in uniform manifold approximation and projection (UMAP) plots of mouse (**e**) and human (**f**) lung scRNA-seq datasets obtained from lungendothelialcellatlas.com. **g,** Primary human lung microvasculature endothelial cell (HMVEC-L) cultures were treated with AHR agonist FICZ or antagonist CH-223191 for 24 hours and indicated gene expression was determined by qPCR (*n* = 6 biological replicates). Statistical analysis was performed using one-way ANOVA with Tukey’s post-test. Data are shown as mean±SEM. Data are shown as mean±SEM. ns, not significant.

**Fig. 1:**
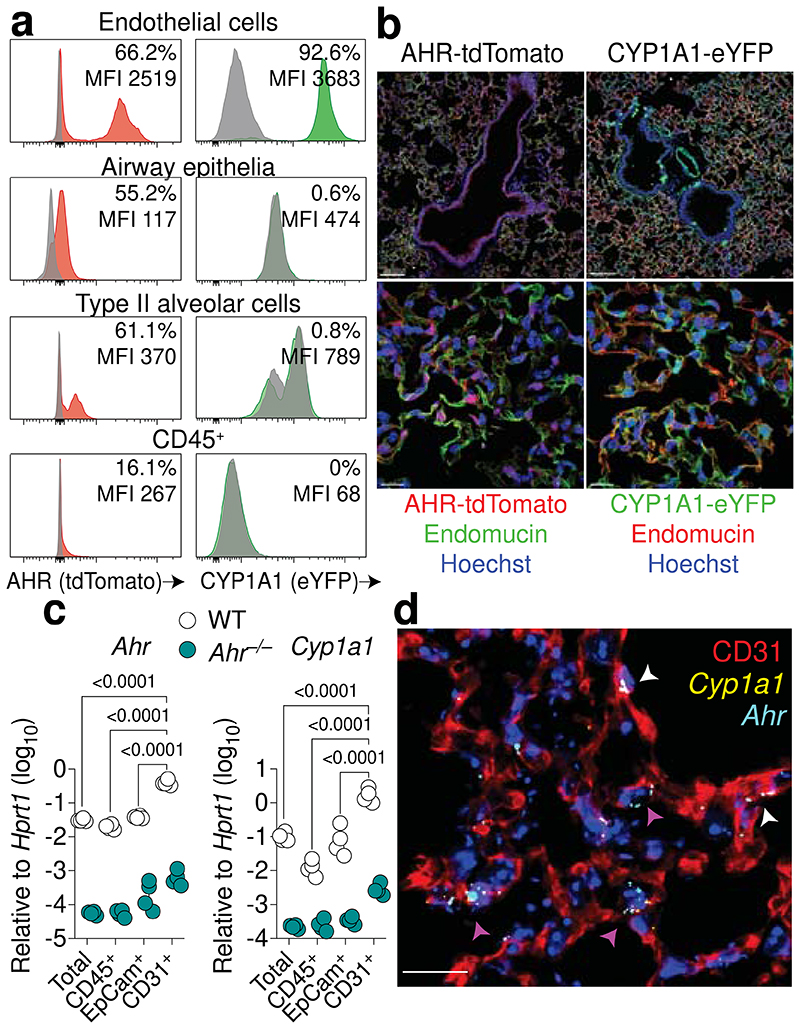
The endothelium is a site of heightened AHR activity in the lung. **a,** Representative histogram plots of AHR-tdTomato and Cyp1a1-eYFP expression in steady-state lung endothelial cells (CD31^+^), airway epithelial cells (EpCam^high^CD24^+^MHC-II^–^), type II alveolar epithelial cells (EpCam^low^MHC-II^+^) and immune cells (CD45^+^) relative to B6 WT controls (grey). Mean fluorescence intensity (MFI). **b,** Overview (upper panels) and maximum intensity projections (lower panels) of Cyp1a1-eYFP and AHR-tdTomato expression in lung sections stained with the endothelial marker endomucin (in green on left panels, red on right panels) and Hoechst (blue). Scale bars, 100 μm (upper panels) and 15 μm (lower panels). **c,** Expression of *Ahr* and target gene *Cyp1a1* in total lung cells, isolated CD31^+^ endothelial cells, EpCam^+^ epithelial cells, and CD45^+^ immune cell populations from WT and *Ahr*^–/–^ mice was determined by qPCR. *n* = 4. **d,** RNA-FISH analysis of WT steady-state lung. RNA probes for *Ahr* (cyan) and *Cyp1a1* (yellow) and stained with CD31 for endothelia. Arrowheads indicate *Ahr/Cyp1a1* expression in alveolar capillary endothelia (purple) or large blood vessels (white). Scale bar, 20 μm. For **c**, data are shown as mean±SEM. Each dot represents an individual mouse. Data are representative of at least two independent experiments with similar results (**a-c**) or four times with similar results (**d**). Statistical analysis was performed using two-way ANOVA followed by Sidak’s post-test (**c**) and significant *P* values are indicated on the graphs.

We took an unbiased systematic approach to characterise AHR expression and signalling in different lung cell compartments by using AHR reporter mice (AHR-tdTomato)^28^ and CYP1A1 fate-reporter mice (CYP1A1Cre/R26R eYFP)^29^. AHR-tdTomato expression was detected in all lung cell types measured, but predominantly in CD31^+^ lung endothelial cells (66.2%), type II alveolar cells (61.1%) and airway epithelial cells (55.2%) (Fig. 1a, Extended Data Fig. 1a). In CYP1A1 fate-reporter mice, AHR activity induces enhanced yellow fluorescent protein (eYFP) expression via a Cre recombinase inserted into the mouse *Cyp1a1* locus^29^. Consistent with the level of AHR-tdTomato expression in endothelial cells, we detected a strong eYFP signal in the lung vasculature (92.6%), yet, to our surprise, sparse activity was recorded in EpCam^+^ lung epithelial cells or CD45^+^ immune cells (Fig. 1a). Furthermore, AHR-tdTomato^+^ endothelia have increased reporter mean fluorescence intensity (MFI), relative to immune cells and epithelia, indicating a higher level of AHR expression. AHR signalling in endothelia was confirmed by immunofluorescence, with both AHR and CYP1A1 fluorescent reporters displaying considerable overlap with endomucin^+^ blood vessels (Fig. 1b). The increased frequency of CYP1A1-eYFP^+^ lung endothelial cells in CYP1A1 fate-reporter mice, relative to AHR-tdTomato^+^, likely reflects prior physiological AHR signalling and Cre-mediated eYFP expression in lung vessels during development, rather than current activity. Both AHR-tdTomato^+^ and CYP1A1-eYFP^+^ cells were detected in lung lymphatic endothelia, but at reduced levels relative to vascular endothelia (Extended Data Fig. 2a, b). In addition, lymphatic endothelia constitute only a small fraction of the total CD31^+^ population (Extended Data Fig. 2c), and therefore likely contribute minimally to lung endothelial AHR activity. To measure active AHR transcription in the mature adult lung, we isolated different lung cell populations and measured AHR and target gene expression by qPCR. Consistent with our findings in reporter mice, isolated CD31^+^ lung endothelial cells displayed significantly increased *Ahr* and *Cyp1a1* expression, relative to lung epithelial cells and immune cells (Fig. 1c). RNA-fluorescence in situ hybridisation (FISH) revealed an AHR signature in the endothelia of both large blood vessels and the alveolar capillary network (Fig. 1d). *Ahr* mRNA was also detected in the airway epithelium, but less so *Cyp1a1* (Extended Data Fig. 2d). Next, we utilised publicly available single-cell RNA sequencing (scRNA-seq) datasets to map the AHR pathway in the lung^30^. AHR expression was detected in multiple lung cell types, including airway epithelia, monocytes, and dendritic cells, and a prominent signature was identified in endothelia, in both mouse and human lungs (Extended Data Fig. 2e, f). *Cyp1a1* expression was almost exclusively detected in endothelial compartments, consistent with our findings using reporter mice or RNA-FISH. Both AHR and CYP1A1 were detected across endothelial cell subsets, with the strongest signal identified in aerocytes, capillaries, and venous endothelia. To confirm AHR activity in human endothelial cells, we established primary human lung microvasculature endothelial cell (HMVEC-L) cultures. HMVEC-L cultures treated with the AHR agonist 6-formylindolo(3,2-b)carbazole (FICZ) induced AHR target gene expression (Extended Data Fig. 2g). Furthermore, treatment with the AHR antagonist CH-223191 resulted in a significant downregulation in *CYP1A1* expression, indicating high basal levels of AHR signalling in primary human lung endothelia.

**Fig. 2:**
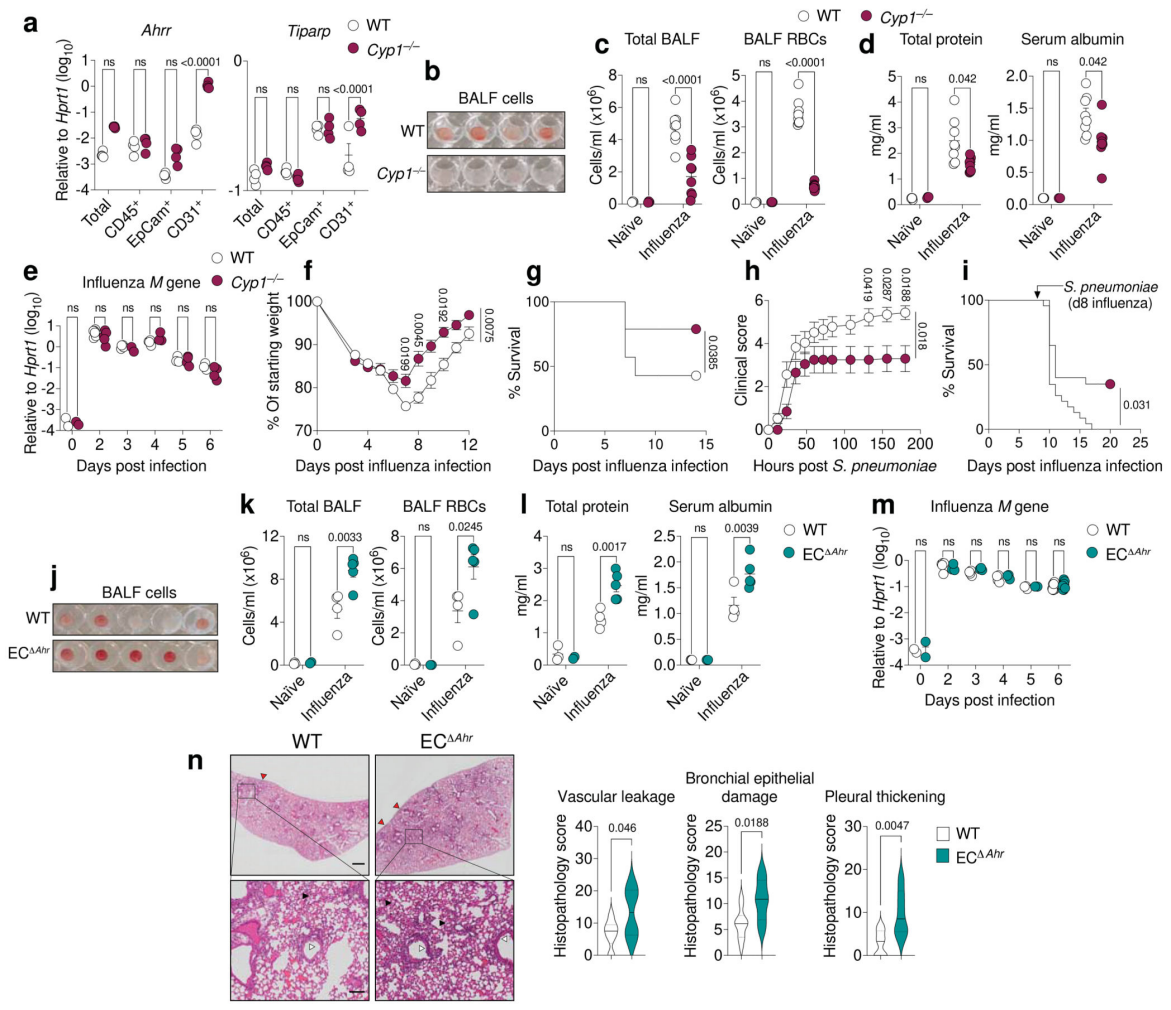
AHR signalling in endothelia prevents lung vascular leakage upon viral infection. **a,** Expression of indicated AHR target genes in FACS-isolated lung cell populations was determined by qPCR (*n* = 4). **b-d, j-l,** Lung damage was assessed in the bronchoalveolar lavage fluid (BALF) of naïve (*n* = 3) and influenza virus infected WT and *Cyp1*^–/–^ mice (*n* = 8 for each genotype) (**b-d**), or WT (*n* = 4) and EC^Δ*Ahr*^ (*n* = 5) mice (**j-l**) by photograph of centrifuged BALF (**b, j**), quantification of total cells and Ter119^+^ RBC counts (**c, k**), and total protein and serum albumin concentrations (**d, l**). (**e, m**) Influenza virus Matrix gene mRNA expression in total lung RNA from naïve and infected WT, *Cyp1*^–/–^ mice (**e**) and EC^Δ*Ahr*^ mice (**m**) was measured by qPCR at indicated days post infection (*n* = 3–9 for each day). **f, g,** Weight loss (**f**) and survival (**g**) of influenza virus infected WT (*n* = 14) and *Cyp1*^–/–^ mice (*n* = 19). **h, i,** Clinical scores (**h**) and survival (**i**) of influenza and *Streptococcus pneumoniae* coinfected WT (*n* = 23) and *Cyp1*^–/–^ (*n* = 20) mice. **n,** Histopathological analysis of hematoxylin and eosin (H&E) lung sections on day 6 after infection (WT *n* = 10, EC^Δ*Ahr*^ n = 6). Black arrowheads indicate signs of lung vascular leakage, red arrowheads indicate areas of pleural thickening, and white arrowheads indicate areas of bronchial epithelial damage. Scale bars, 500 μm (upper panels) and 100 μm (lower panels). All Data are representative of two to four independent experiments with similar results or pooled from three experiments (**f-i**). Statistical analysis was performed using two-way ANOVA with Sidak’s post-test (**a, c-f, h, k-m**), log-rank (Mantel-Cox) test (**g, i**), or two-tailed Mann–Whitney *U* test (**n**) and significant *P* values are indicated on the graphs. Data are shown as mean±SEM. ns, not significant.

We next asked whether increasing AHR ligand availability would augment AHR signalling in lung cell types in vivo. To assess this, we utilised mice deficient for the three CYP1 family enzymes (*Cyp1a1*, *Cyp1b1* and *Cyp1a2*) regulated by AHR^31^. *Cyp1*^–/–^ mice are unable to metabolise AHR ligands and therefore lack autoregulatory feedback control, resulting in an expanded pool of available natural ligands and constitutive AHR signalling^16,17,21^. CYP1 deficiency led to increased expression of AHR target genes (*Ahrr* and *Tiparp*) in CD31^+^ lung endothelial cells, with only a modest increase in epithelia and no change in immune cells (Fig. 2a). Taken together, these results identify the vascular endothelium as a site of heightened AHR activity in both mouse and human lung.

## AHR protects against lung vascular leakage

**Extended Data Fig. 3:**
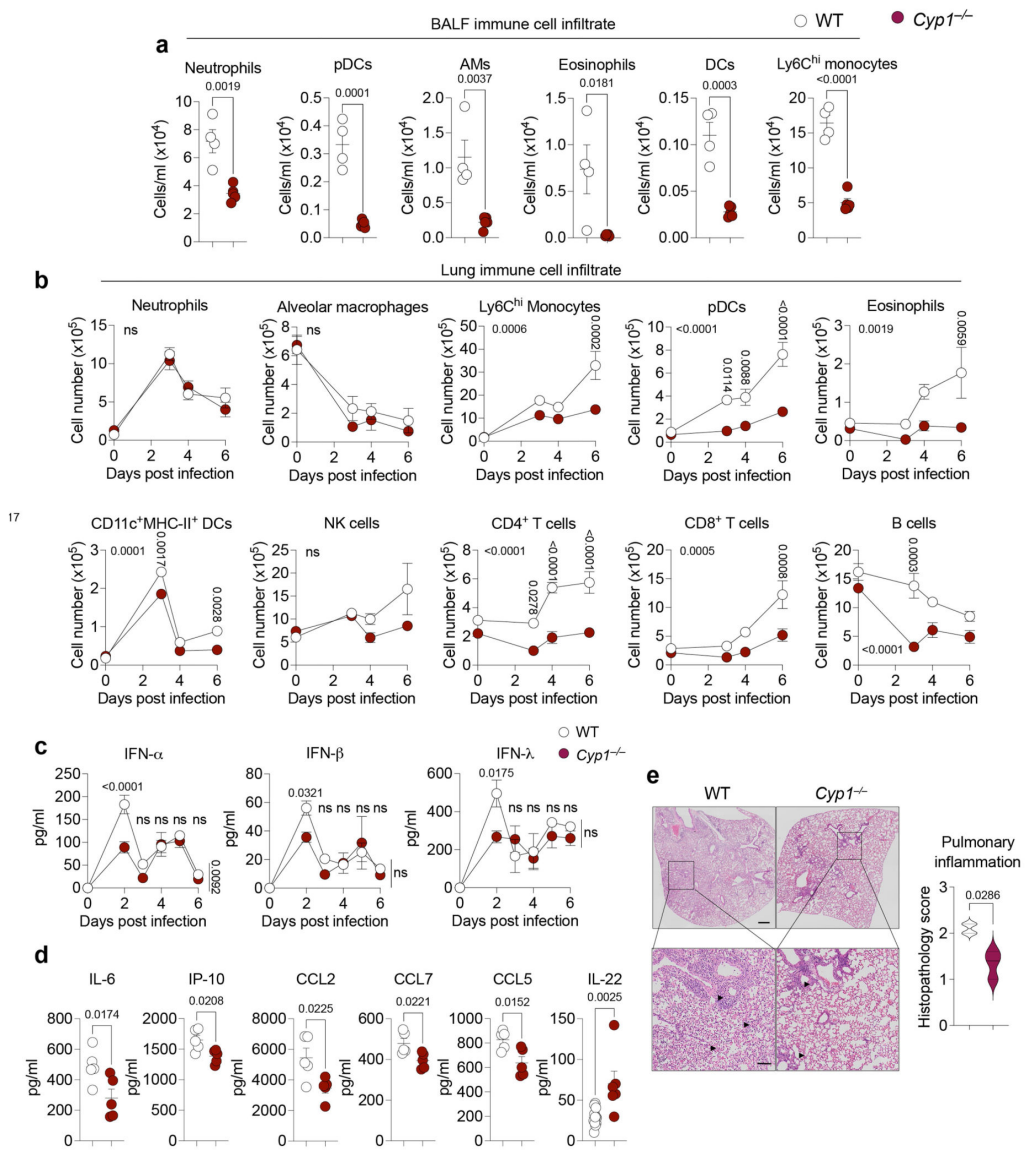
Dampened pulmonary inflammation in CYP1-deficient mice. **a, b,** Lung immune cell numbers were determined in the BALF on day 6 post infection (**a**) or in whole lung on indicated days post infection (**b**) in WT (*n* = 4) and *Cyp1*^–/–^ (*n* = 5) mice by flow cytometry. **c, d,** BALF cytokine concentration in influenza virus infected WT and *Cyp1*^–/–^ mice was determined on indicated days for IFN (*n* = 3) (**c**) or day 6 for remaining cytokines (**d**) post infection (*n* = 5). **e,** Histopathological analysis of WT (*n* = 4) and *Cyp1*^–/–^ (*n* = 3) H&E lung sections on day 6 post infection. Black arrowheads indicate areas of perivascular, peribronchiolar, and intra-alveolar inflammatory cell infiltration. Scale bars, 500 μm (upper panels) and 100 μm (lower panels). All Data are representative of three to four independent experiments. Statistical analysis was performed using unpaired two-tailed Student’s *t* test (**a, d**), two-way ANOVA with Sidak’s post-test (**b, c)**, or two-tailed Mann–Whitney *U* test (**e**) and significant *P* values are indicated on the graphs. Data are shown as mean±SEM. ns, not significant.

**Extended Data Fig. 4:**
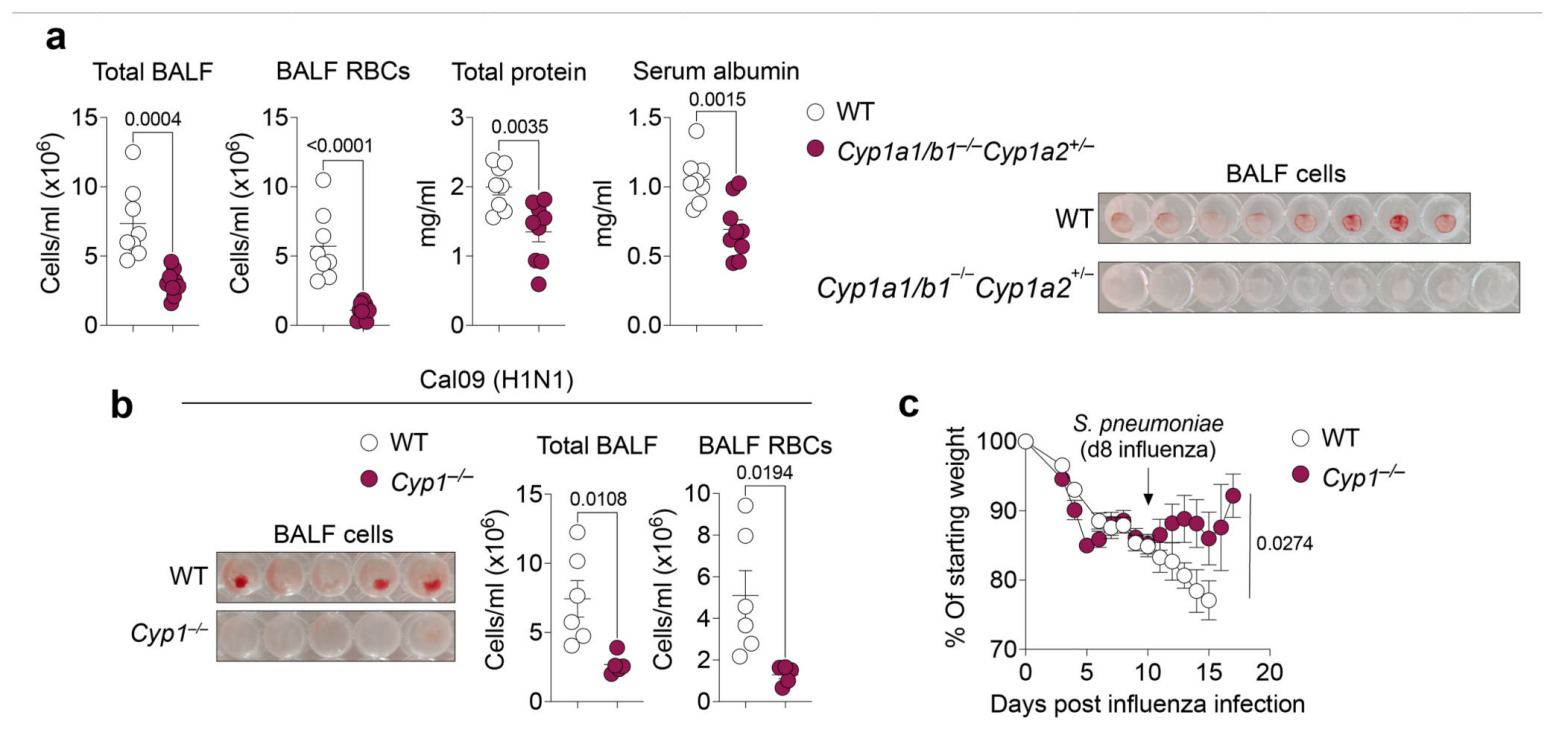
CYP1 deficiency confers protection against respiratory pathogens. **a, b,** Lung damage was assessed in the BALF of X31 influenza virus infected WT (*n* = 8) and *Cyp1a1/Cyp1b1* double-knockouts (*Cyp1a2*^+/-^) (*n* = 9) (**a**) or Cal09 H1N1 influenza virus infected WT (*n* = 6) and *Cyp1*^–/–^ (*n* = 5) mice (**b**) by measurement of total cells, Ter119^+^ RBCs, total protein, and serum albumin concentrations on day 6 post infection. (**c**) Weight loss of influenza (X31) and *Streptococcus pneumoniae* coinfected WT (*n* = 23) and *Cyp1*^–/–^ (*n* = 23) mice. All Data are representative of two independent experiments or pooled from three experiments (**c**). Statistical analysis was performed using unpaired two-tailed Student’s *t* test (**a**, **b**) or two-way ANOVA with Sidak’s post-test (**c**) and significant *P* values are indicated on the graphs. Data are shown as mean±SEM.

To understand whether amplified AHR signalling in endothelial cells affects lung barrier function, we infected WT and *Cyp1*^–/–^ mice with influenza A virus (X31) and assessed lung damage. By analysing the bronchoalveolar lavage fluid (BALF) on day 6 post influenza virus infection, we found that *Cyp1*^–/–^ mice were more resistant to infection-induced lung barrier leakage, with reduced total cells, red blood cells (RBCs), and immune cell infiltration within the lung airspace, relative to wild-type (WT) controls (Fig. 2b, c, Extended Data Fig. 1b, Extended Data Fig. 3a). Reduced cellular infiltration was consistent with reduced markers of lung damage, measured by total protein and serum albumin concentrations in the BALF of influenza infected *Cyp1*^–/–^ mice (Fig. 2d). Increased lung protection in *Cyp1*^–/–^ mice occurred without changes in viral burden (Fig. 2e), indicating that protection associated with increased AHR signalling is due to enhanced disease tolerance mechanisms instead of altered antiviral immunity^32^. Decreased lung damage in *Cyp1*^–/–^ mice was consistent with reduced morbidity and increased survival upon infection (Fig. 2f, g). We also detected a significantly dampened proinflammatory response in *Cyp1*^–/–^ mice upon influenza virus infection, characterised by reduced levels of pulmonary immune cell recruitment, reduced early IFN production, lower concentrations of proinflammatory cytokines and chemokines in the BALF, and reduced immune cell distribution in the lung parenchyma (Extended Data Fig. 3b-e). Mice deficient for *Cyp1a1* and *Cyp1b1*, but heterozygous for *Cyp1a2*, whose expression and functional importance is largely restricted to the liver^33^, also displayed increased resistance to influenza-induced vascular leakage and lung damage (Extended Data Fig. 4a), indicating that the increased protection conferred by CYP1 deficiency is unrelated to altered CYP1A2-driven ligand metabolism in the liver. To understand whether augmented AHR signalling promotes protection against other respiratory pathogens, we infected WT and *Cyp1*^–/–^ mice with an antigenically distinct influenza virus variant, Cal09 (H1N1), a representative strain of the 2009 “swine flu” pandemic. CYP1-deficient mice had reduced cellular infiltration and vascular leakage in lung air spaces upon Cal09 infection (Extended Data Fig. 4b).

Furthermore, *Cyp1*^–/–^ mice also showed reduced weight loss, developed lower clinical scores, and had improved survival rates following infuenza-*Streptococcus pneumoniae* co-infection (Fig. 2h, i, and Extended Data Fig. 4c). These data highlight the protective effects conferred upon ablation of AHR negative feedback regulation in promoting lung barrier integrity, thus reducing disease severity upon infection by diverse respiratory pathogens.

**Extended Data Fig. 5:**
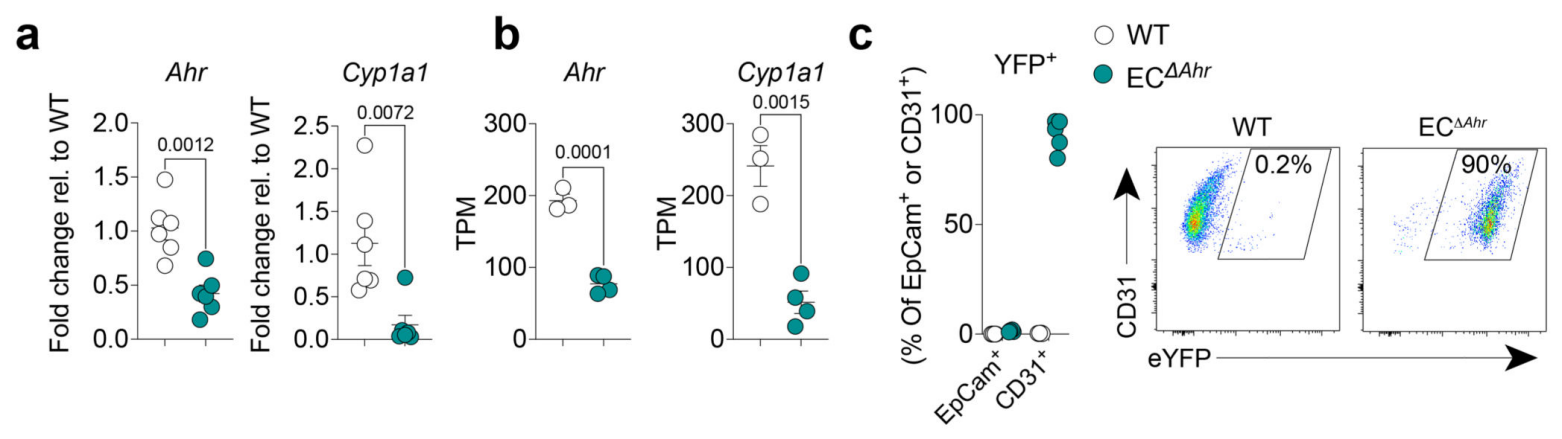
Endothelial-specific AHR deletion. **a,** Endothelial-specific AHR deletion was determined by measuring expression of *Ahr* and AHR-target gene *Cyp1a1* in isolated lung CD31^+^ endothelial cells in *Cdh5*^Cre-ERT2^*Rosa26*-LSL-YFP; *Ahr*^flox/flox^ mice (EC^Δ*Ahr*^) and WT control (*Cdh5*^Cre-^*Rosa26*-LSL-YFP; *Ahr*^flox/flox^) mice by qPCR (*n* = 8) (**a**) or transcripts per million (TPM) from bulk RNA-seq analysis (*n* = 3) (**b**). **c,** YFP-expressing lung endothelial cells as a measurement of Cre induction was determined in CD31^+^ lung endothelial cells by flow cytometry in EC^Δ*Ahr*^ mice (*n* = 5). All Data are representative of two independent experiments. Statistical analysis was performed using unpaired two-tailed Student’s *t* test and significant *P* values are indicated on the graphs. Data are shown as mean±SEM.

**Extended Data Fig. 6:**
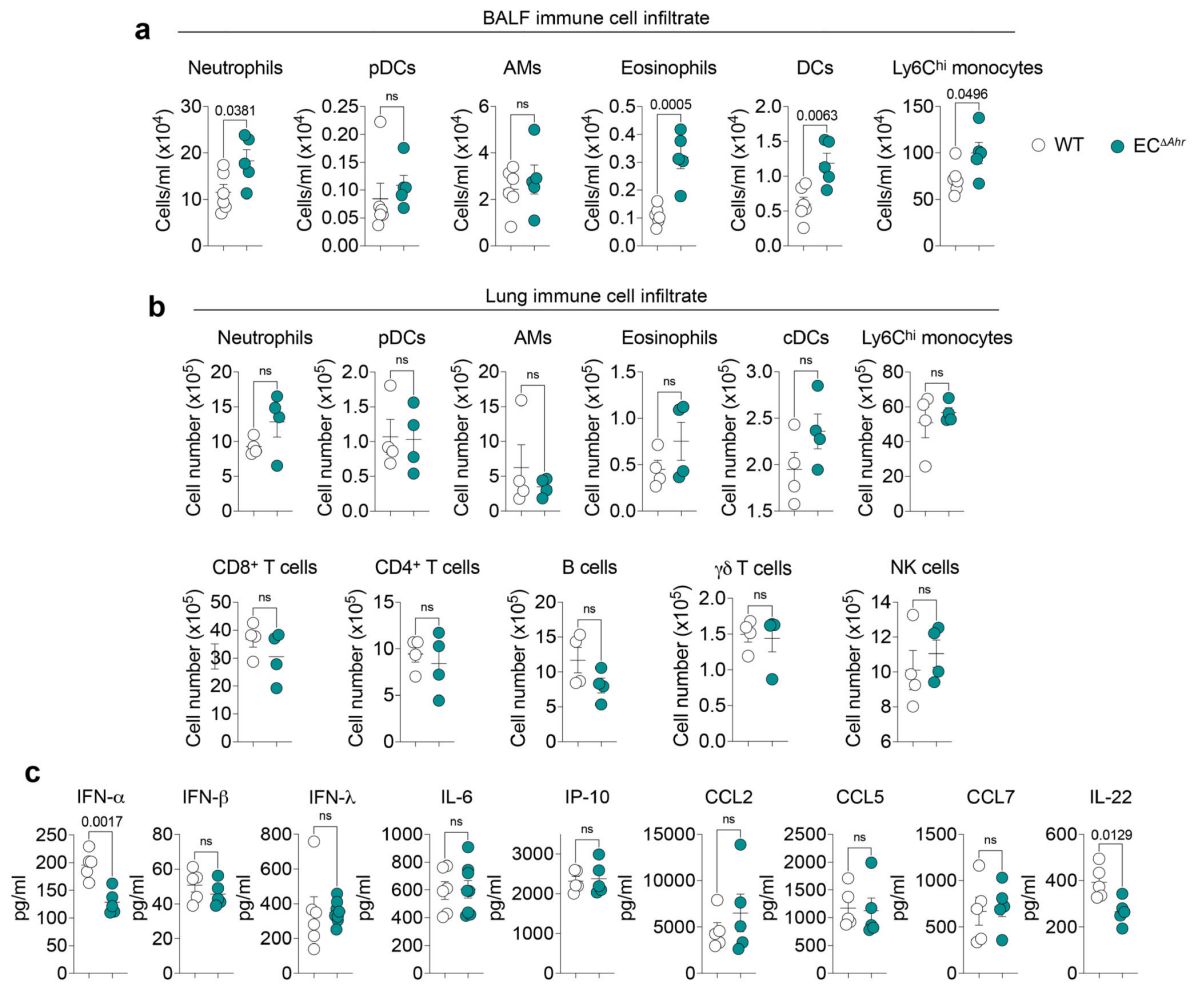
AHR deletion in endothelial cells does not drastically alter influenza-induced pulmonary inflammation. **a, b** Immune cell numbers were determined in the BALF of WT (*n* = 6) and EC^Δ*Ahr*^ (*n* = 5) mice (**a**) and whole lung (*n* = 4) (**b**) of on day 6 post infection by flow cytometry. **c,** BALF cytokine concentration in WT and EC^Δ*Ahr*^ mice was determined on day 2 (IFN) or day 6 (remaining cytokines) post infection (IL-6 and IFN-λ: WT *n* = 6, EC^Δ*Ahr*^ *n* = 8; remaining cytokines *n* = 4). All Data are representative of two to three independent experiments. Statistical analysis was performed using unpaired two-tailed Student’s *t* test and significant *P* values are indicated on the graphs. Data are shown as mean±SEM. ns, not significant.

We next investigated the importance of physiological AHR signalling in endothelial cells in promoting lung barrier protection upon viral challenge. To study this, we used *Cdh5*^Cre-ERT2^*Rosa26*-LSL-YFP; *Ahr*^flox/flox^ mice (EC^Δ*Ahr*^), in which tamoxifen treatment induces Cre recombinase activity and excision of the loxP flanked *Ahr* allele specifically in *Cdh5* (VE-Cadherin) expressing endothelial cells. Tamoxifen treatment efficiently reduced *Ahr* expression and activity in lung endothelial cells (Extended Data Fig. 5). Consistent with the enhanced protection observed in *Cyp1*^–/–^ mice, tamoxifen-induced endothelial *Ahr* deletion caused increased lung vascular leakage, exacerbated tissue damage, and promoted cellular infiltration in the airspace upon influenza virus infection, relative to Cre^–^ littermate controls (Fig. 2j-l, Extended Data Fig. 6a). Increased lung damage in EC^Δ*Ahr*^ mice was independent of alterations in influenza viral burden (Fig. 2m). However, AHR-deletion in endothelial cells did not significantly alter the wider pulmonary inflammatory landscape (Extended Data Fig. 6b, c). Histopathological assessment of lung tissue confirmed an increase in alveolar space fluid leakage, highlighting significant barrier disruption in influenza infected EC^Δ*Ahr*^ mice, in addition to bronchial epithelial damage and thickening of the lung pleura (Fig. 2n). Taken together, these results identify endothelial-specific AHR activity as a host mechanism of lung barrier protection which prevents vascular leakage in air spaces upon viral infection.

## Endothelial protection of the respiratory epithelium

**Extended Data Fig. 7:**
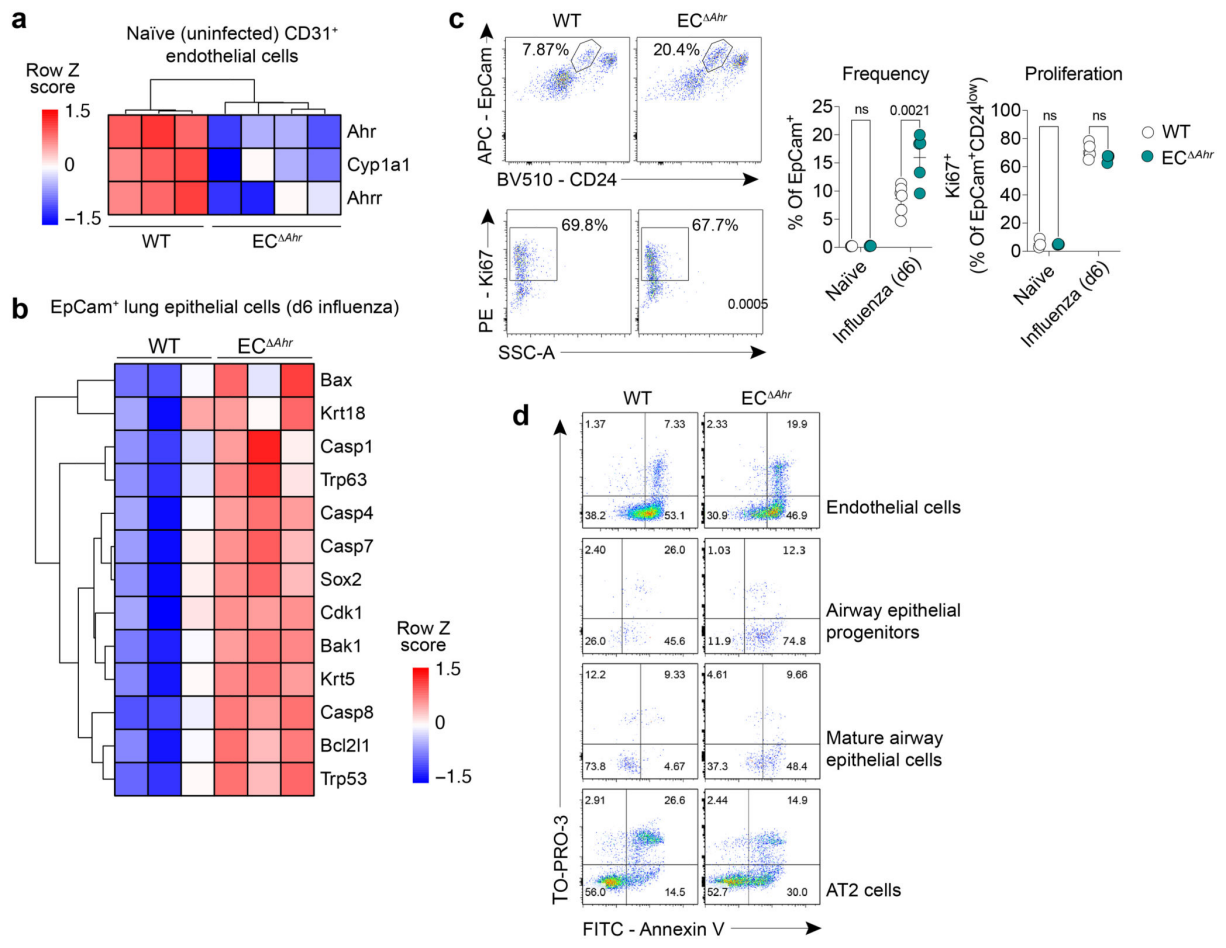
AHR signalling in endothelia prevents airway epithelial apoptosis and dysplastic repair. **a, b,** Heatmaps of indicated genes from bulk RNA sequencing data comparing naïve WT and EC^Δ*Ahr*^ CD31^+^ lung endothelial cells (**a**) and EpCam^+^ lung epithelial cells on day 6 post infection (**b**) (fold change > 1.5, *padj* < 0.05). **c,** Frequency (% of total EpCam^+^) and proliferation (Ki67^+^) of distal airway stem cells (EpCam^high^CD24^low^MHC-II^–^) in the lungs of WT and EC^Δ*Ahr*^ mice was measured by flow cytometry in naïve (*n* = 3) mice and on day 6 post influenza infection (WT *n* = 6; EC^Δ*Ahr*^ *n* = 5). **d,** Frequency of apoptotic (Annexin-V^+^) and necrotic (TO-PRO-3^+^) lung endothelial cells (CD31^+^), progenitor airway epithelial cells (EpCam^high^CD24^low^MHC-II^–^), mature airway epithelial cells (EpCam^high^CD24^high^MHC-II^–^), and type II alveolar epithelial cells (EpCam^low^MHC-II^+^) was assessed by flow cytometry in WT and EC^Δ*Ahr*^ mice on day 6 post influenza infection (*n* = 4). All Data are representative of two independent experiments. Statistical analysis was performed using one-sided Wald test with Benjamini–Hochberg correction (**a, b)** or two-way ANOVA with Sidak’s post-test (**c**) and significant *P* values are indicated on the graphs. Data are shown as mean±SEM. ns, not significant.

**Fig. 3:**
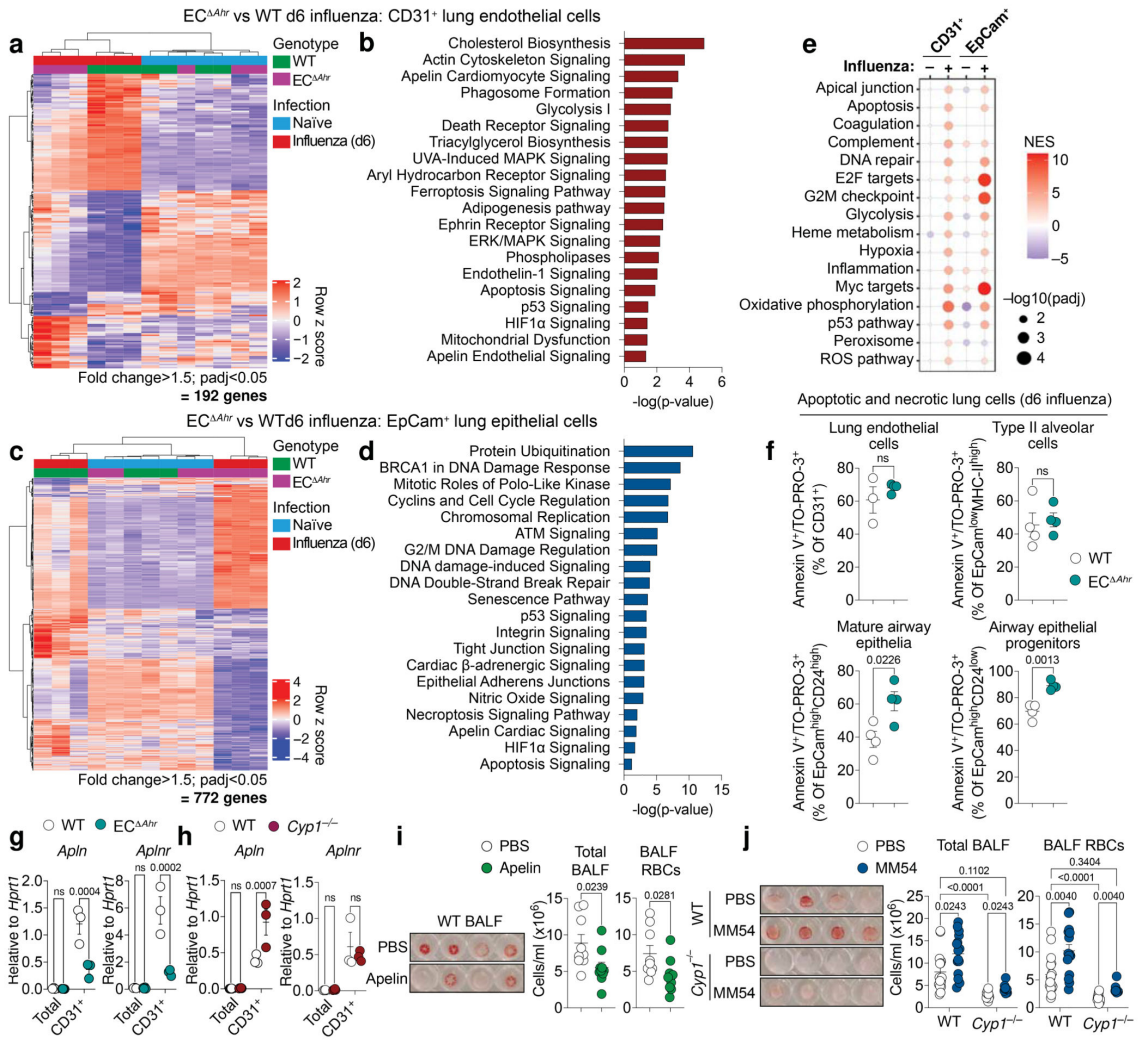
Endothelial-AHR mediates lung protection via apelin signalling and prevents a dysplastic apoptotic response in airway epithelia. **a, c, Heatmaps showing** differentially expressed genes in lung endothelia (**a**) or epithelia (**c**) comparing influenza infected WT and EC^Δ*Ahr*^ mice. **b, d,** Selected canonical pathways differentially regulated between infected WT and EC^Δ*Ahr*^ lung endothelial cells (**b**) and epithelial cells (**d**) using ingenuity pathway analysis (IPA) (*padj* < 0.05, fold change > 1.5). (**e**) Dot plot of hallmark pathways in EC^Δ*Ahr*^ mice (relative to WT) using GSEA (four pairwise comparisons total). Dot size relates to statistical significance. (**f**) Frequency of apoptotic (Annexin-V^+^) and necrotic (TO-PRO-3^+^) lung endothelia (CD31^+^), progenitor airway epithelial cells (EpCam^high^CD24^low^MHC-II^–^), mature airway epithelia (EpCam^high^CD24^high^MHC-II^–^), and type II alveolar epithelial cells (EpCam^low^MHC-II^+^) was assessed by flow cytometry in influenza infected WT and EC^Δ*Ahr*^ mice (*n* = 4). **g, h,** *Apln* and *Aplnr* expression was determined in total lung and lung endothelial cells isolated from influenza infected WT, EC^Δ*Ahr*^ (**g**) and *Cyp1*^–/–^ (**h**) (*n* = 3). **i, j,** Lung vascular leakage was determined in PBS (*n* = 9) or apelin (*n* = 10) treated WT mice (**i**) and PBS or MM54-treated WT (PBS *n* = 16; MM54 *n* = 15) and *Cyp1*^–/–^ (PBS *n* = 11; MM54 *n* = 10) mice (**j**) by quantification of total cell and RBCs. All Data are representative of two to three independent experiments with similar results or pooled from three experiments (**j**). Statistical analysis was performed using a one-sided Wald test with Benjamini–Hochberg correction (**a-d**), normalised enrichment scores (NES) were generated with GSEA using a two-sided Kolmogorov Smirnov statistic with Hallmark genesets on genelists ranked by the Wald t statistic from DESeq2 (**e**) unpaired two-tailed Student’s *t* test (**f, i**), or two-way ANOVA with Sidak’s post-test (**g, h, j**) and significant *P* values are indicated on the graphs. Data are shown as mean±SEM. ns, not significant.

To understand mechanistically how endothelial-specific AHR activity mediates protection in the lung, we performed bulk RNA-sequencing on pulmonary endothelial cells (CD31^+^) isolated from naïve and influenza virus infected WT and EC^Δ*Ahr*^ mice (day 6 post infection). Apart from the expected downregulation in AHR signalling (Extended Data Fig.7a), we detected minimal further disruption in the expression profile of endothelial cells isolated from naïve EC^Δ*Ahr*^ mice, relative to WT control (Fig. 3a). Indeed, endothelial cells from uninfected mice clustered together irrespective of genotype. However, upon influenza virus infection, endothelial samples clustered separately according to genotype due to a significant divergence in expression profiles (Fig. 3a), indicating that protective AHR signalling becomes evident upon lung challenge. Pathway analysis of differentially expressed genes in infected WT versus EC^Δ*Ahr*^ endothelial cells identified significant disruption in cellular stress responses, metabolism, and cell death, in the absence of endothelial AHR (Fig. 3b, Table S1).

In parallel, we profiled the transcriptome of lung epithelial cells (EpCam^+^) in naïve and influenza infected WT and EC^Δ*Ahr*^ mice to understand indirect consequences of disrupted endothelial AHR signalling. Endothelial-specific *Ahr* deletion had almost no effect on lung epithelial cell gene expression in steady state conditions (5 differentially expressed genes; padj < 0.05). However, upon infection, we detected widespread disruption in the transcriptional profile of epithelial cells from EC^Δ*Ahr*^ mice (Fig. 3c). Pathway analysis revealed marked dysregulation in proliferation and cell-cell signalling in EC^Δ*Ahr*^ epithelial cells (Fig. 3d, Table S2), suggesting an extensive AHR-driven cross talk between the lung endothelium and epithelium following viral infection. Gene Set Enrichment Analysis (GSEA) confirmed enrichment of a cellular stress profile (DNA repair, hypoxia, OxPhos, p53, ROS), evidence of barrier disruption and vascular leakage (apical junction, apoptosis, coagulation, complement), and inflammatory signalling (hypoxia, inflammation, glycolysis) in EC^Δ*Ahr*^ endothelia, as well as an aberrant mitotic profile in damaged epithelial cells (Fig. 3e, Table S3).

Of notable interest was the increased expression of apoptotic genes and a dysplastic keratinised signature in lung epithelial cells isolated from EC^Δ*Ahr*^ mice (Extended Data Fig. 7b). This includes *Krt5* and *Trp63* expression; markers of a distal airway stem cell subset which expand in damaged tissue regions following severe alveolar loss, giving rise to keratinised ‘pods’ that compromise lung gas exchange function^34–36^. Indeed, we detected an increased frequency of EpCam^high^CD24^low^ distal airway stem cells in the lungs of influenza infected EC^Δ*Ahr*^ mice (Extended Data Fig. 7c). This damage-associated epithelial subset^37,38^ is almost entirely absent in the naïve lung, is highly proliferative, and although no differences in the rate of proliferation was detected, its increased expansion in infected EC^Δ*Ahr*^ lungs likely contributes to the dysregulated mitotic epithelial signature (Fig. 3d, e) and epithelial disruption as evidenced by histology (Fig. 2n). Furthermore, we found a significant increase in the frequency of apoptotic and necrotic airway epithelial cells upon influenza virus infection, however, no differences were detected in endothelial cells or type II alveolar cells (Fig. 3f, Extended Data Fig. 7d). Overall, these findings highlight the importance of AHR signalling in the endothelium upon lung injury. AHR activity in the vasculature prevents endothelial stress and barrier disruption, while also tempering a dysplastic keratinised and apoptotic signature in airway epithelial cells following influenza virus infection.

**Extended Data Fig. 8:**
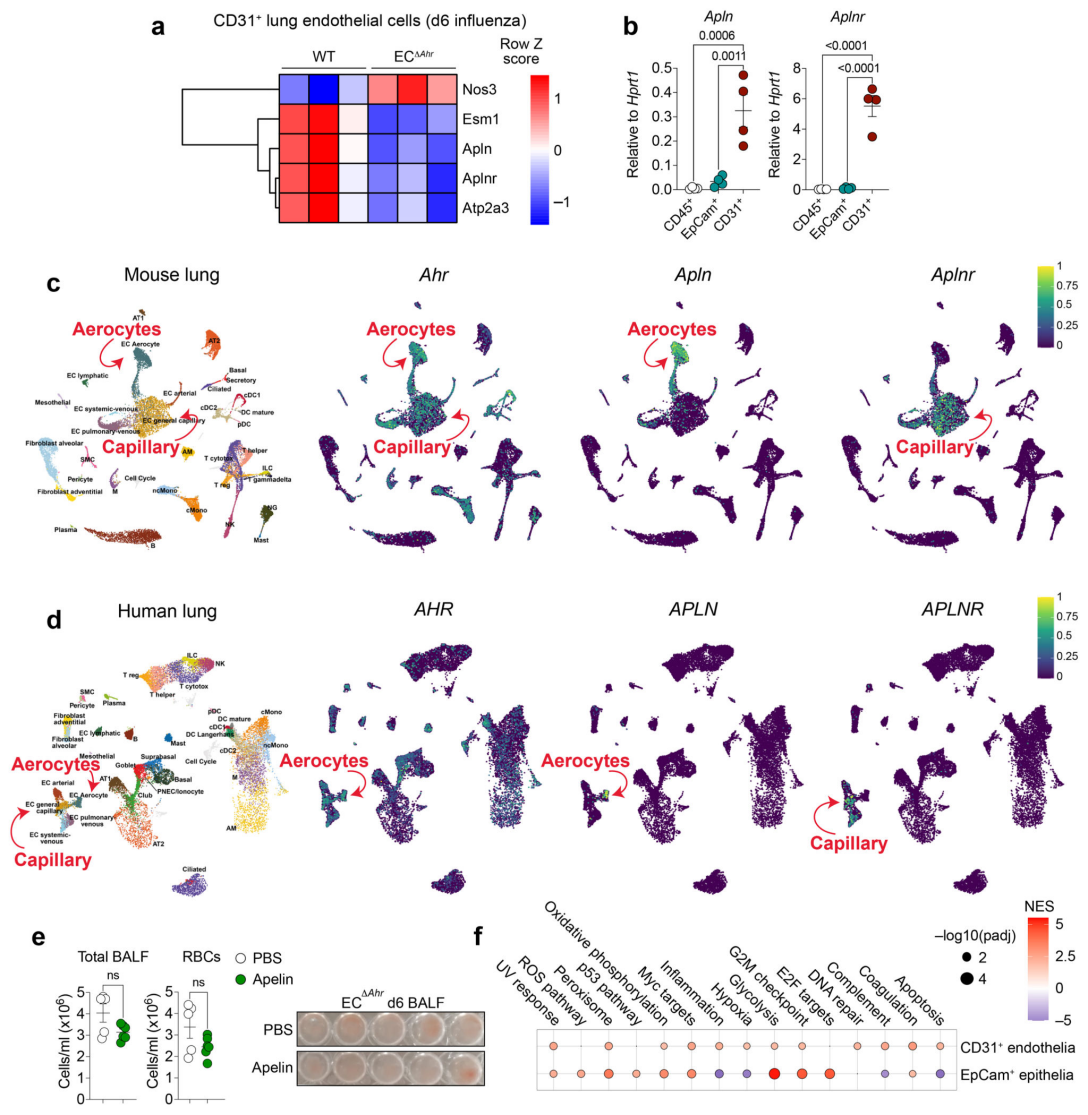
AHR-dependent regulation of the apelin signalling pathway in lung endothelia. **a,** Heatmap of indicated genes from RNA sequencing data comparing CD31^+^ lung endothelial cells on day 6 post infection (fold change > 1.5, *padj* < 0.05). **b,** Expression of indicated genes in isolated CD45+ immune cell, EpCam^+^ epithelial cell, and CD31^+^ endothelial cell was determined by qPCR in naïve WT mice (*n* = 4). **c, d,** Expression of indicated genes in UMAP plots of mouse (**c**) and human (**d**) lung scRNA-seq datasets obtained from lungendothelialcellatlas.com. **e,** Lung vascular leakage was assessed in PBS (*n* = 5) and apelin (*n* = 6) treated EC^Δ*Ahr*^ mice by quantification of total cells Ter119^+^ RBCs in the BALF on day 6 post infection. **f,** Dot plot of hallmark pathways enriched or downregulated in MM54-treated WT mice (relative to PBS-treated controls) by GSEA. Comparisons are between MM54-treated and untreated from influenza infected mice, for endothelia and epithelia (two pairwise comparisons total). Dot size relates to statistical significance. All Data are representative of at least two independent experiments. Statistical analysis was performed using one-sided Wald test with Benjamini–Hochberg correction (**a**) or followed by Tukey’s post-test (**b**), or unpaired two-tailed Student’s *t* test (**e**). NES were generated with GSEA using a two-sided Kolmogorov Smirnov statistic with Hallmark genesets on genelists ranked by the Wald t statistic from DESeq2 (**f**) and significant *P* values are indicated on the graphs. Data are shown as mean±SEM. ns, not significant.

Among the most significantly disrupted pathways in EC^Δ*Ahr*^ endothelial cells was apelin signalling (Fig. 3b, Extended Data Fig. 8a). Apelin — a vasoactive endogenous peptide produced by endothelial cells — is involved in the regulation of vessel function through the apelin receptor (APLNR)^39^. The expression of both *Apln* and its receptor, *Aplnr*, is restricted to endothelial cells in the lung (Extended Data Fig. 8b). This was confirmed by analysis of public scRNA-seq datasets^30^, which revealed that *Apln* and *Aplnr* are expressed in aerocytes and capillary endothelia, respectively (Extended Data Fig. 8c, d), consistent with previous reports^40^. Importantly, we detected a remarkable degree of overlap between AHR and the apelin system in both human and murine lung endothelia. We confirmed disruption of apelin and apelin receptor expression in EC^Δ*Ahr*^ endothelial cells by qPCR (Fig. 3g). Consistently, CYP1-deficient mice which have increased AHR signalling in lung endothelia also displayed increased *Apln* expression in the vasculature (Fig. 3h). Therapeutic apelin delivery has previously been shown to prevent tissue damage in acute lung injury^41–43^. To test whether apelin treatment protects against influenza-induced lung vascular leakage, we administered exogenous apelin to infected WT and EC^Δ*Ahr*^ mice. Apelin treatment significantly reduced lung damage and vascular leakage in influenza virus infected WT mice (Fig. 3i), but not in EC^Δ*Ahr*^ mice (Extended Data Fig. 8e). We reasoned that the reduced efficacy of apelin application in EC^Δ*Ahr*^ mice was due to their marked decrease in vessel *Aplnr* expression (Fig. 3g). Thus, we decided to pharmacologically block APLNR in *Cyp1*^–/–^ mice to understand whether increased AHR signalling mediates protection via positive regulation apelin signalling. Both WT and *Cyp1*^–/–^ mice exhibited increased lung vascular leakage following treatment with the competitive APLNR antagonist, MM54^44^ (Fig. 3j). Furthermore, pharmacological blockade of apelin signalling in WT mice caused an enrichment of pathways relating to endothelial cell stress and vascular leakage in endothelia, as well as disrupted mitotic signalling and remodelling in epithelia (Extended Data Fig. 8f, Table S4), comparable to the expression profile observed in EC^Δ*Ahr*^ lungs (Fig. 3b). Taken together, these findings identify the apelin/APLNR signalling axis as a mechanism of AHR-dependent lung protection, preventing endothelial stress, vascular leakage, and epithelial remodelling, upon influenza-induced lung damage.

## A gut-lung axis determines lung pathology

**Fig. 4:**
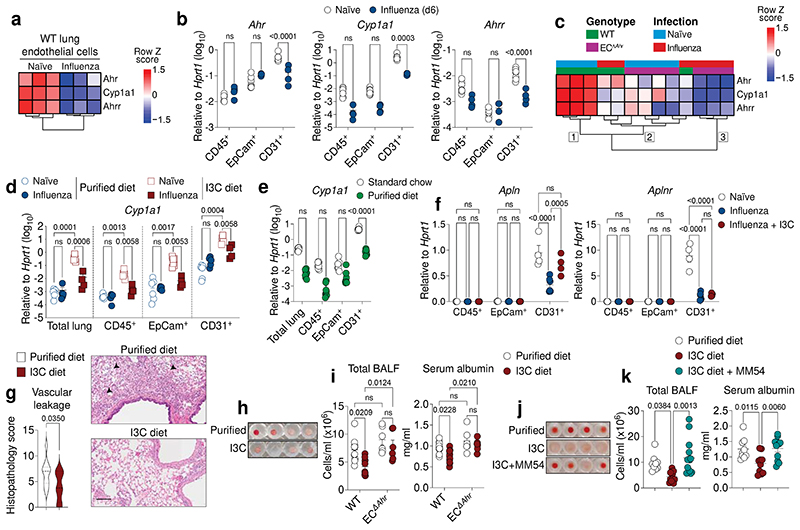
Loss of protective lung AHR signalling upon influenza virus infection is regulated by dietary intake. **a, c,** Heatmap of indicated genes from bulk RNA-seq data comparing lung endothelial cells from naïve and influenza infected WT (**a**) or WT and EC^Δ*Ahr*^ mice (**c**). Numbers indicate the three main dendogram clusters (**c**). **b, d-f,** Expression of indicated genes in total lung, CD31^+^ endothelial, EpCam^+^ epithelial, and CD45^+^ immune cell populations determined by qPCR in naïve (*n* = 7) and influenza infected (*n* = 4) WT mice (**b**), WT naïve (*n* = 7) and infected (*n* = 4–5) mice fed purified or I3C diet (**d**), WT naïve mice fed purified diet (*n* = 8) or standard chow (*n* = 7) (**e**), in naïve (*n* = 4) or infected (*n* = 6) mice fed purified diet or infected mice fed I3C diet (*n* = 4) (**f**). **g**, Histopathological analysis of H&E lung sections from influenza infected WT mice fed purified or I3C-enriched diet (*n* = 10). Black arrowheads indicate signs of vascular leakage. Scale bar, 100 μm. **h-k,** Lung damage assessed in the BALF of influenza infected WT (*n* = 10) and EC^Δ*Ahr*^ (*n* = 6) mice fed purified or I3C diet (**h, i**) and WT mice fed purified diet (*n* = 9), I3C diet (*n* = 11) or I3C diet with MM54 treatment (*n* = 14) (**j, k**) by photograph of total BALF cells (**h, j**) and quantification of total cells and serum albumin concentrations in BALF (**i, k**). All Data are representative of at least two independent experiments with similar results or pooled from two experiments (**i, k**). Statistical analysis was performed using one-sided Wald test with Benjamini–Hochberg correction (**a, c)**, one-way ANOVA with Dunnett’s post-test (**k**), two-way ANOVA with Sidak’s post-test (**b, d-f, i),** or two-tailed Mann–Whitney *U* test (**g**) and significant *P* values are indicated on the graphs. Data are shown as mean±SEM. ns, not significant.

A highly consistent feature in our influenza model was the marked depression of AHR signalling in WT lung endothelial cells upon infection (Fig. 4a, b). Considering the protective role AHR plays in lung vessels (Fig. 2k–n), we hypothesised that infection-induced perturbation of the AHR response may dampen protective signalling in endothelia. While suppression of AHR signalling may exacerbate lung damage, it is important to note that protective AHR signalling in lung endothelial cells is not completely lost following infection, as infected EC^Δ*Ahr*^ mice exhibit further decreased AHR signalling (Fig. 4c and Extended Data Fig. 5) and have increased lung vascular leakage (Fig. 2j, k), relative to infected WT controls. Indeed, unsupervised hierarchical clustering stratifies WT and EC^Δ*Ahr*^ endothelial samples into three main clusters according to infection status and level of AHR activity: 1) uninfected WT (strongest AHR signature), 2) infected WT and uninfected EC^Δ*Ahr*^ (dampened AHR signature), and 3) infected EC^Δ*Ahr*^ (weakest AHR signature) (Fig. 4c). These findings highlight the extent of AHR pathway suppression in lung endothelial cells upon influenza virus infection.

We next sought to understand what signals induced by infection cause such a decrease in lung endothelial AHR activity. Dietary metabolites are a rich source of natural AHR ligands^15^. We speculated whether suppression of lung AHR signalling upon infection was caused by alterations in dietary intake or metabolism. To test this, we increased AHR ligand intake through dietary supplementation with indole-3-carbinol (I3C), a proligand which is converted to high affinity agonists by stomach acids^45^, and compared this to mice fed a purified control diet starved of dietary indoles. Mice fed an I3C-enriched diet had significantly increased AHR signalling in all lung cell subsets measured, but predominantly in lung endothelial cells (Fig. 4d). Interestingly, influenza virus infection suppressed AHR signalling induced by the AHR ligand-rich I3C diet, with no observable difference in AHR activity detected in naïve and influenza virus infected mice fed purified diet which lacks AHR ligands (Fig. 4d). Furthermore, purified control diet had reduced AHR signalling in lung endothelia compared to mice fed standard animal house chow (Fig. 4e), highlighting the contribution of natural dietary ligands to lung AHR activity. *Apln* expression was also decreased in lung endothelia following influenza infection, which could be restored by supplementing the diet with AHR ligands (Fig. 4f). Taken together, these findings show that protective AHR activity in endothelial cells is regulated by dietary intake, which can be modified by infection status.

**Extended Data Fig. 9:**
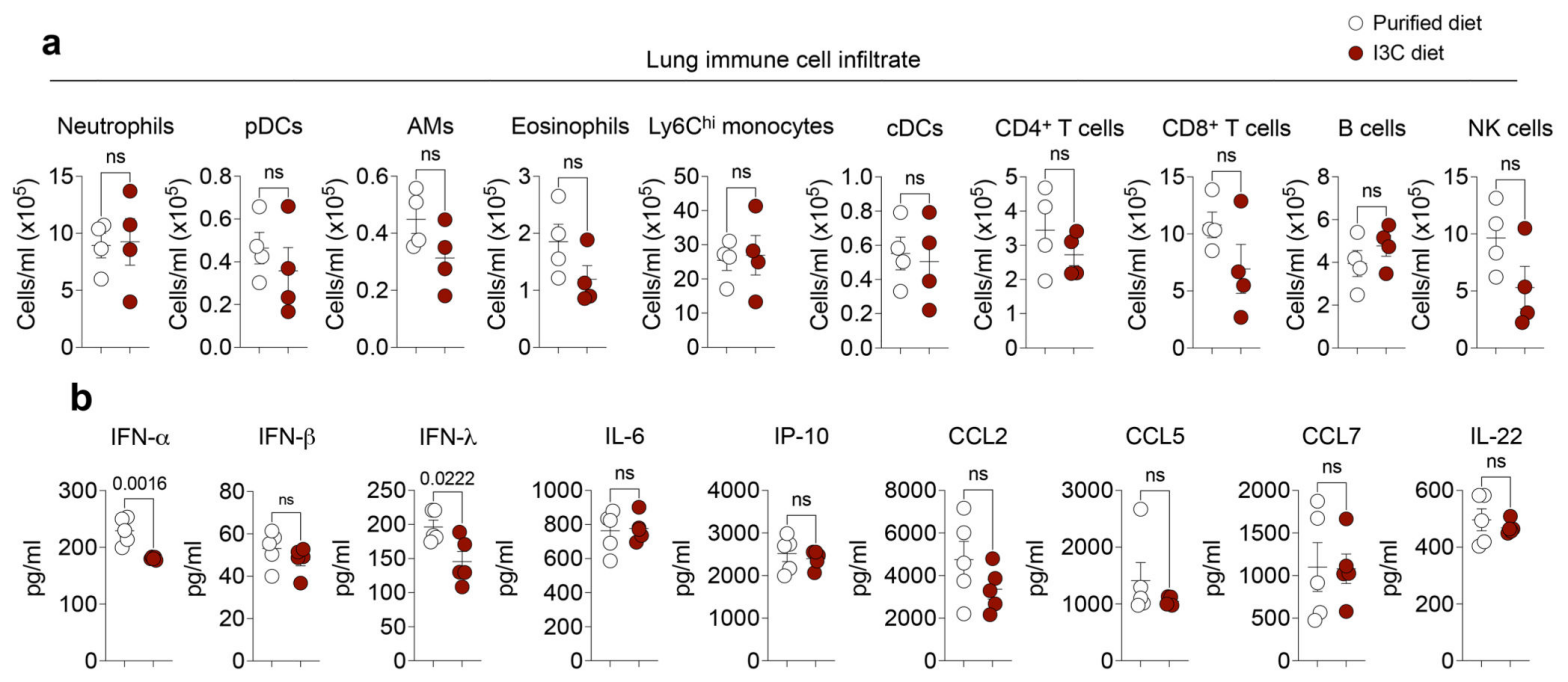
Dietary AHR ligands do not disrupt pulmonary inflammation. **a,** Immune cell numbers were determined in the whole lung of WT mice fed purified or I3C-enriched diet on day 6 post infection by flow cytometry (*n* = 4). **b,** BALF IFN (day 2) and cytokine (day 6) concentrations in WT mice fed purified or I3C-enriched diet (*n* = 5). Statistical analysis was performed using unpaired two-tailed Student’s *t* test and significant *P* values are indicated on the graphs. Data are shown as mean±SEM. ns, not significant.

We next sought to understand whether augmented AHR signalling mediated by I3C-supplemented diets affected disease progression. Mice fed the I3C-supplemented diet exhibited similar levels of immune cell recruitment, cytokine, and chemokine production, except for a small decrease in type I and III IFN production (Extended Data Fig. 9a, b), consistent with the suppression of IFN production following AHR activation (Extended Data Fig. 3c)^25^. However, amplified lung AHR signalling induced by dietary ligands protected mice against influenza-induced tissue damage and vascular leakage (Fig. 4g-i). I3C diet-induced lung protection was dependent on both AHR signalling in endothelial cells (Fig. 4h, i) and apelin receptor signalling (Fig. 4j, k). Overall, these findings show that natural dietary AHR ligands induce protective AHR signalling in the lung vasculature along a gut-lung axis to promote host defence against respiratory viral pathogens.

## Discussion

A crucial component of the host response to infection is the induction of tissue-protective disease tolerance programmes which function to limit the deleterious impact of the infection itself and of the ensuing inflammatory immune response^32^. Here we identify AHR signalling in the lung endothelium as an active mechanism of disease tolerance during respiratory virus infection. We show minimal AHR-dependent regulation of endothelial function in the steady-state lung. Only upon viral infection is the wider regulatory capacity of endothelial AHR function realised, suggesting that in addition to ligand binding, AHR-driven expression programmes can be regulated by secondary signals derived from the disruption of lung homeostasis. This is in line with the quiescence of the lung tissue in steady-state, including endothelia, which becomes activated upon insult to dampen tissue damage and promote repair^9–13^. Regulation of AHR vascular function will also likely be influenced by the tissue-specific microenvironment. The gastrointestinal tract, for instance, boasts a rich supply of microbial- and dietary-derived AHR ligands at a site with extensive steady-state cellular turnover, which may provide the signals required to engage barrier-supportive vascular AHR function. Indeed, the accompanying study by Wiggins *et al*. demonstrates that AHR sensing of dietary ligands in enteric endothelia promotes barrier homeostasis and the induction of anti-inflammatory pathways, comparable to what we observe in the lung following viral infection.

We identify an additional layer of regulation underlying AHR lung function with the observation that protective AHR signalling is significantly depressed following viral infection. We show that host intake of naturally occurring dietary indoles derived from precursors rich in cruciferous vegetables^45,46^ amplify AHR-apelin signalling in the respiratory endothelium to prevent lung tissue damage. Interestingly, we find diet-dependent lung AHR activity to be dampened by the infection itself, suggesting that infection-induced alterations in dietary metabolism or consumption affect lung function via AHR. For instance, mice exhibit reduced feeding and increased weight loss following influenza virus infection (Fig. 2f), which may restrict the supply of natural dietary AHR ligands to the lung, ultimately compromising lung barrier integrity through a loss of protective AHR signalling in endothelial cells.

The lung endothelium operates as a primary interface on two fronts: 1) as a barrier to the outside environment in conjunction with the respiratory epithelium; and 2) as a conduit to the rest of the body via the circulatory system. The potential for perturbation of the AHR response through these interfaces, for instance dietary metabolites trafficking in the blood or environmental pollutants, links AHR to inter-individual clinical variability in respiratory viral disease severity. While we identify the endothelium as a site of heightened AHR activity in the lung, we do not exclude roles for other cell types in regulating AHR-dependent lung function^26^. Indeed, AHR is activated in both the lung epithelium and immune cell compartment following dietary I3C supplementation (Fig. 4d), albeit to a lesser extent than in endothelial cells. While AHR function at barrier surfaces has been extensively studied in epithelial cells and immune cells^47^, our study highlights the importance of endothelial-specific AHR function in promoting host resistance to influenza virus infection. We also reveal a prominent interplay between the lung endothelium and epithelium, mediated by endothelial-specific AHR activity, which functions to prevent a marked mitotic, apoptotic, and dysplastic epithelial response upon viral challenge. These findings highlight the broader implications of AHR-endothelial regulation on the lung tissue architecture in respiratory viral disease.

The heightened responsivity of endothelial cells in the lung to dietary I3C suggests that endothelial function at other distal organs may also be affected by diet-dependent regulation of AHR activity. This may have implications for other types of infection or for pathological inflammatory diseases. For instance, pharmacological AHR activation is clinically beneficial in the treatment of psoriasis^48^, and psoriatic lesions have decreased AHR signalling in endothelia relative to healthy controls^49^. Therefore, disease outcome may be altered by endothelial responsivity to natural AHR ligands in the diet. Future studies may also look to explore the effects of non-AHR dietary metabolites on distal tissue endothelial cell function in homeostasis and in disease contexts.

In summary, our findings identify the lung endothelium as a key player in coordinating the tissue response to acute respiratory viral infection via AHR activity. Therapeutic intervention strategies targeting the AHR-apelin axis or lifestyle modifications relating to balanced dietary intake may prove effective in ameliorating lung damage clinically^50^.

## Methods

### Mice

All animal experiments were approved by the Home Office, UK, under project license P9C468066 and carried out in accordance with the Animals (Scientific Procedures) Act 1986. All experiments used male and female mice at 6–14 weeks of age, bred at the Francis Crick Institute (2016–2022) under specific pathogen-free conditions. For in vivo experiments, mice were age- and sex-matched where possible, between genotypes and treatment groups. All genetically modified mice were bred on a C57BL/6J background and maintained as homozygous lines. Genotypes used for in vivo experiments were C57BL/6J, AHR-tdTomato^28^, *Cyp1a1-eYFP* fate-reporter mice (CYP1A1Cre; R26R eYFP)^21^, *Cyp1a1*^–/–^*Cyp1a2*^–/–^*Cyp1b1*^–/–^ (*Cyp1*^–/–^) triple knockouts^31^, *Cyp1a1^–/–^Cyp1b1^–/–^Cyp1a2*^+/–^ double knockouts, and *Cdh5*^Cre-ERT2^*Rosa26*-LSL-YFP; *Ahr*^flox/flox^ mice (EC^Δ*Ahr*^).

### Influenza viruses

X31 and Cal09 (gifts from J. Skehel, MRC-NIMR) were grown in the allantoic cavity of 10-day-embryonated hen’s eggs and were free of bacterial, mycoplasma, and endotoxin contamination. All viruses were stored at −80°C and titrated on Madin–Darby canine kidney cells. Virus was quantified in infected lungs by qPCR for the influenza *Matrix* gene on cDNA from whole lungs, normalized to the housekeeping gene *Hprt1*.

### Infections

C57BL/6J, *Cyp1*^–/–^, and EC^Δ*Ahr*^ mice were infected with X31 (35,000 TCID_50_ in 30 μl of PBS), or Cal09 (10,000 TCID50 in 30 μl of PBS). For co-infections mice were infected with 5,000 TCID50 X31 in 30 ul of PBS and 8 days later infected with *Streptococcus pneumoniae* (TIGR4) (2.5x10^5^ c.f.u. in 30 gl PBS). All infections were performed under light anesthesia (3% isoflurane) intranasally. Pre-infection body weights were recorded, and mice were weighed daily (at a consistent time of the day) and monitored for clinical symptoms. Mice reached the clinical endpoint following the loss of 25% of their initial starting weight or at a clinical score ≥5. Clinical scores were determined by (1 point each) piloerection, hunched posture, partially closed eyes, laboured breathing, hypothermia, decreased movement,movement only on provocation or (2 points) absence of movement on provocation or (5 points) middle-ear infection (disrupted balance).

### In vivo treatments

WT and EC^Δ*Ahr*^ mice were administered apelin ([Pyr1]-Apelin-13, Sigma) at a dose of 650 nmol/kg intraperitoneally every 24 hours on day 2 to 5 post influenza virus infection. WT and *Cyp1*^–/–^ mice were administered the APLNR antagonist, MM54 (Tocris Bioscience), intraperitoneally at a dose of 4 mg/kg on days −1, 0, 2, and 4 post infection. For diet studies mice were fed purified diet AIN-93M or AIN-93M supplemented with 1000 p.p.m. indole-3-carbinol (Ssniff). Mice were put on purified diets shortly after weaning for at least 4 weeks and maintained on the purified diets throughout experiments.

### Quantification of damage and cytokines in BALF

Mice were euthanized (500 mg/kg Ketamine + 50 mg/kg Xylazine) and BALF was collected through catheter insertion intratracheally, injecting and retracting 1 ml of ice-cold PBS. BALF was centrifuged at 300 × *g* for 5 min at 4°C and supernatants were stored at −80°C. Pelleted cells in the BALF were counted and analysed by flow cytometry to determine the frequency of Ter119^+^ RBCs on day 6 post infection unless otherwise indicated. Frequencies of immune cell populations were quantified by flow cytometry. Concentrations of IFN-α/β were measured using 2-Plex ProcartaPlex (Invitrogen). IFN-λ was measured by ELISA (R&D). Total protein concentrations in BALF were determined using the Pierce BCA Protein Assay Kit (Thermo Scientific) according to manufacturer’s instructions. Serum albumin was quantified using a BCG (Bromocresol Green) Albumin Assay Kit (Sigma) according to manufacturer’s instructions. The ProcartaPlex Cytokine and Chemokine Mouse 36-Plex (eBioscience) was used to assess cytokine concentrations in BALF read on a Luminex 100 (BioRad).

### Flow cytometry

For isolation of cell populations from lung tissue, mice were euthanized (500 mg/kg Ketamine + 50 mg/kg Xylazine) and then perfused with 10 ml of ice-cold PBS through the right ventricle of the heart. Dispase II (5 mg/ml in AB-IMDM) (Sigma) was then injected intratracheally (1.5 ml) into the lungs, followed by 0.4 ml 0.8% low-gelling agarose solution (in PBS) (Sigma). Mice were placed on ice allowing the agarose–dispase-filled lungs to set. Lungs were dissected and placed in 2 ml of dispase II solution and shaken gently for 30 min at room temperature to dissociate endothelial cells and epithelial cells. Lungs were passed through a 100μm filter prior to a 10-minute DNase I digestion (50 μg/ml) (Sigma). Following digestion, lung homogenates were passed through a 70 μm filter and centrifuged at 300 × *g*. for 5 min at 4°C, before red blood cell lysis. Single-cell suspensions were preincubated with anti-FcgRIII/II (Fc block), before a 30-min incubation with one or more fluorochrome-labelled antibodies. The following antibodies and staining reagents were used: EpCam-APC, (17-5791-82) 2.5 μg/ml; CD45-BV786 (103149) 2.5 μg/ml; CD31-BV421 (103149) 2.5 μg/ml; CD24-BV510 (101831) 10 μg/ml; MHC-II-BV711 (107643) 2 μg/ml; Ly6C-BV786, (128041) 5 μg/ml; Ly6G-FITC (127605) 10 μg/ml; CD3-APC (20-0032-U025) 20 μg/ml; CD3-FITC (35-0032-U025) 10 μg/ml; CD19-APC (115511) 20 μg/ml; NK1.1-APC (108709) 20 μg/ml; NK1.1-PE (108707) 20 μg/ml; CD64-PeCy7 (139313) 20 μg/ml; Siglec F-APC-Cy7 (BDB565527) 3.3 μg/ml; CD11b-Pacific Blue (101223) 10 μg/ml; CD11c-BV605 (117333) 5 μg/ml; CD4-BV605 (100547) 5 μg/ml; CD8a-BV510 (100751) 5 μg/ml; TCRgd-eFluor 450 (48-5711-82) 5 μg/ml; Siglec H-PE (129605) 10 μg/ml; Ter119-BV510 (116237) 10 μg/ml; PDPN-APC-Cy7 (127417) 20 μg/ml; Annexin V-FITC (640905) 10 μg/ml; Fixable blue dead stain-BUV395 (L23105) 1 μl/10^6^ cells. Annexin V staining (eBioscience) was performed as per manufacturer’s instructions and dead cells were identified by TO-PRO-3 (ThermoFisher) staining. Cells were analysed on a BD LSRFortessa cell analyser (BD Bioscience) and interpreted using the software FlowJo v10.6.2 (FlowJo, RRID:SCR_008520).

### RNA isolation

For total lung RNA, lungs were harvested and stored in RNALater in -80°C. Lungs were homogenised with a Kinematica Polytron PT 10-35 homogenizer in 3 ml of RLT buffer (QIAGEN) + β-mercaptoethanol. RNA was isolated using the Qiagen RNeasy mini kit, according to the manufacturer’s instructions. 200 ng of total RNA was reverse-transcribed using the qPCRBIO cDNA synthesis kit (PCR Biosystems) as per manufacturer’s instructions. RT-qPCR was performed on an Applied Biosystems Quantstudio 3 RT-qPCR machine with 1X qPCRBIO Probe Mix Lo-ROX (PCR Biosystems), and 1X Taqman primers (Life Technologies). The following probes were used: *Ahr* (Hs00169233_m1, Mm00478930_m1), *Ahrr* (Mm00477443_m1), *Apln* (mm00443562_m1), *Aplnr* (mm00442191_s1), *Cyp1a1* (Hs01054796_g1, Mm00487218_m1), *Hprt1* (Mm00446968_m1), *Tiparp* (Mm00724822_m1). Primers for influenza virus *Matrix* gene were as follows:

forward: 5’-AAGACCAATCCTGTCACCTCTGA-3’

reverse: 5’-CAAAGCGTCTACGCTGCAGTCC-3’

probe: 5’-TTTGTGTTCACGCTCACCGT-3’

For the isolation of lung cell subsets, tissue digests were blocked with Fc block in MACS (magnetic-activated cell sorting) buffer prior to incubation with biotinylated anti-CD45. Cells were subsequently incubated with streptavidin microbeads (Miltenyi Biotech) and separated using an LS column (Miltenyi Biotech) on the magnetic field of a MACS Separator (Miltenyi Biotech) to isolate CD45^+^ immune cells. CD45^+^ lung immune cells were lysed in 350 μl of RLT buffer + β-mercaptoethanol. From CD45-depleted lung cell digests, endothelial cells (live single cells, CD31^+^EpCam^–^CD45^–^) and epithelial cells (live single cells, EpCam^+^CD31^–^CD45^–^) were FACS-isolated using a FACS Aria III (BD Biosciences) and lysed in RLT buffer + β-mercaptoethanol.

### RNA sequencing

RNA-sequencing was performed on the HiSeq 4000 system (Illumina) with Single End 75 bp reads. Sequencing yield was ~25 million strand specific reads per sample. Read quality trimming and adaptor removal was carried out using Trimmomatic (version 0.36). Reads were aligned to the mouse genome (Ensembl GRCm38 release 89) using STAR (version 2.5.2a)^51^ and gene level counts were obtained using the RSEM package (version 1.3.0)^52^. For RSEM, all parameters were run as default except “-forward-prob” that was set to 0.5. Differential expression analysis was carried out with DESeq2 package (version 1.24.0)^53^ within R version 3.6.0^54^. Genes were considered to be differential expressed with an adjusted p value < 0.05. Gene Set Enrichment analysis (GSEA, version 2.2.3)^55^ was performed for each pairwise comparison using gene lists ranked using the Wald statistic. Gene set pre-ranked analysis was carried out using C2 canonical pathways v7.4 and C5 biological processes v7.4. All parameters were kept as default except for enrichment statistic that was changed to classic and the max size which was changed to 500,000. Gene signatures were considered significant if FDR q-value % 0.05. Over enrichment analyses was performed using Qiagen’s IPA software^56^ using differentially expressed genes with a fold change >1.5 and adjusted P value <0.05.

### Histology and immunofluorescence

Whole lungs were inflated with intratracheal injection of 10% neutral buffered formalin (NBF), and fixed overnight also in 10% NBF, then embedded in paraffin and sectioned. Lung specimens were stained with hematoxylin and eosin (H&E) and then subjected to gross and microscopic pathologic analysis. Histopathological analysis was performed by two board-certified veterinary pathologists, blinded to the groupings/genotype, of various parameters as follows. For bronchioles evidence of injury (epithelial erosion (including loss of cilia) or ulceration) and inflammation (intraluminal neutrophils) were assessed. Evidence of vascular leakage within alveolar spaces was recorded as presence of interalveolar haemorrhage, fibrin and/or proteinaceous oedema fluid. Finally, the pleura was assessed for reactive change (mesothelial cell hypertrophy). All parameters were assessed semi-quantitatively for severity, 0; no lesion, 1; minimal change, 2; mild change, 3; moderate change and 4; marked change. Pathology scores were adjusted according to the total percentage of affected tissue.

For immunofluorescence of lung sections, frozen OCT slides were thawed at room temperature for 20 minutes, re-hydrated in PBS and blocked with 3% BSA, 0.3% Triton X-100 in PBS (blocking buffer) for 1 hour at room temperature in a humid chamber. Primary antibodies were diluted in blocking buffer and incubated overnight at 4°C in a humid chamber. TdTomato in AHR-tdTomato direct reporter was detected with an anti-RFP and eYFP in CYP1A1 lineage tracer was detected with a polyclonal anti-eGFP. Primary antibodies were detected with Alexa Flour-conjugated antibodies incubated for 1 hour at room temperature. Nuclei were counterstained with Hoechst. The following antibodies and staining reagents were used: Anti-RFP (600-401-369) 1/600; Anti-Endomucin (sc-65495) 1/100; Anti-CD31 (AF3628) 1/50 dilution; Anti-E-cadherin (20874-1-AP) 1/2000; Anti-GFP (Ab290) 1/1000 dilution; anti-rabbit AF488 (A21206) 1/400; anti-rat AF594 (A21209) 1/400; anti-rabbit AF555 (A31572) 1/400; anti-rat AF488 (A21208) 1/400.

### RNA-FISH

5 μm FFPE sections were stained on the Leica Bond Rx automated stainer using RNAscope™ LS Multiplex Fluorescent assay (ACD Bio-Techne) applying a standard 15 min target retrieval and 15 min protease treatment using target probes *Cyp1a1* (464618) and *Ahr* (452098-C4). Following RNAscope samples were immunostained with CD31 (AF3628 Biotechne) or E-cadherin (20874-1-AP Proteintec). Opal 570, Opal 620 and Opal 690 TSA fluorophores (Akoya Biosciences) were used and imaged on the PhenoImager® HT (Akoya Biosciences). Subsequent spectral unmixing and background subtraction was performed using Akoya Biosciences inForm® software which relies on the use of a library of emission spectra for the Opal fluorophores. Images were analysed using QuPath and ImageJ.

### Primary human endothelial cell cultures

Human biological samples were sourced ethically, and their research use was in accord with the terms of the informed consents. Human lung microvasculature endothelial cells (HMVEC-L) were obtained from Lonza (Cat no. cc-2527). Human endothelial cell cultures were grown at 37°C in Endothelial Cell Growth Media 2 (PromoCell) with 5% CO_2_. Cultures were treated with the AHR agonist FICZ (Enzo, 250 nM) or AHR antagonist CH-223191 (Selleck, 3 μM) for 24 hours. Endothelial cells were lysed in 350 μl of RLT + β-mercaptoethanol for RNA extraction.

### Statistical analysis

Data shown as the mean ± SEM. Sample sizes were designed to give statistical power, while minimizing animal use. All statistical comparisons were performed using Prism 9 (GraphPad). Figure legends denote the specific statistical tests used for each experiment. Statistical significance was determined as *P*<0.05.

## Extended Data

## Supplementary Material

Table S1

Table S2

Table S3

Table S4

## Data Availability

Sequencing data are available in GEO under accession codes GSE203427 and GSE225958.
